# Effects of screen exposure on young children’s cognitive development: A review

**DOI:** 10.3389/fpsyg.2022.923370

**Published:** 2022-08-17

**Authors:** Bahia Guellai, Eszter Somogyi, Rana Esseily, Adrien Chopin

**Affiliations:** ^1^Département de Psychologie, Université Paris Nanterre, Nanterre, France; ^2^Department of Psychology, Faculty of Science and Health, University of Portsmouth, Portsmouth, United Kingdom; ^3^Institut de la Vision, Sorbonne Université, INSERM, CNRS, Paris, France

**Keywords:** screens, cognitive development, prevalence, context, quality, language

## Abstract

The past decade has witnessed a rapid increase in the use of screen media in families, and infants are exposed to screens at younger ages than ever before. The objective of this review is twofold: (1) to understand the correlates and demographic factors determining exposure to screens, including interactive screens, when available, and (2) to study the effects of watching screens and using touchscreens on cognitive development, during the first 3 years of life. We argue that the effects of screen viewing depend mostly on contextual aspects of the viewing rather than on the quantity of viewing. That context includes the behavior of adult caregivers during viewing, the watched content in relation to the child’s age, the interactivity of the screen and whether the screen is in the background or not. Depending on the context, screen viewing can have positive, neutral or negative effects on infants’ cognition.

## Introduction

Over the past 30 years, the number of television programs targeting infants has been increasing, resulting in infants spending more time watching screens and an earlier exposure (Chen and Adler, 2019). For example, by using time diary data from 1997 and 2014 Child Development Supplement of the Panel Study of Income Dynamics, Chen and Adler (2019) show that between 1997 and 2014, screen time doubled among children aged 0 to 2 years. Christakis (2009) reported that the average age of first exposure to television was at 4 months. Given the rapid increase of exposure to screens and at a very early age, in 1999, the American Academy of Pediatrics (AAP) recommended that children under the age of 2 should not be exposed to screens (American Academy of Pediatrics, 1999). These recommendations were followed by numerous studies over the next 10 years showing that screen exposure in children under 3 years of age can be both harmful and beneficial for their cognitive development, depending on the context in which viewing occurs (i.e., content of the program, parents’ investment in program choice, commenting while children watch screens, screen interactivity and screen in the background). In 2011, after the release of the first interactive screens, the AAP reiterated their recommendations despite the fact that only few studies exist so far on the effects of these new types of screens on infants’ development (American Academy of Pediatrics, 2011). In their 2016 statement, the AAP addressed a series of concrete recommendations for parents and caregivers to develop a family media plan. For example, they recommend co-viewing with their parents for young infants and to limit screen use to qualitative programs for only an hour per day for older children (American Academy of Pediatrics, 2016).

The purpose of this article is to understand which kind of screen exposure is harmful for cognitive development and whether some viewing contexts can be beneficial for learning in infants under 3 years of age. Indeed, most of the articles on the subject have not considered the importance of the context of exposure. Here, we will refer to television and mobile devices generally as screens and indicate the type of screen discussed where relevant. In particular, we will review content available about the effect of touchscreens.

In the present narrative review, we propose to highlight the possible links between screen exposure and young children’ cognitive development. We selected articles in the last two decades related to the effect of media on the child’s cognition, focusing more on the early childhood period that is likely to be the most susceptible to any effect of screens, and excluding entries linked to the effects of violence in the media or in video games on the child’s emotions. To reflect these choices, we used Google Scholar as an academic search engine through the software “Publish or Perish,” with the following keywords in the title: “infant,” or “child,” or “children,” or “toddler,” or “childhood,” or “development,” and “television,” or “screen,” or “media,” or “video”; excluding those with the keyword “games” in the title, and including the keywords “early” or “young,” and “cognition,” while excluding the keywords: “autism,” “screening,” “otitis,” “emotion,” “violence,” “prize.” This allowed us to select 478 unique peer-reviewed articles between January 1, 2000 and August 2, 2020, and 102 of which we review here, selected according to the additional criteria below.

We decided to focus on the most studied cognitive areas at that age, therefore excluding studies not focused on the effects on language development, executive functions, imitation, parent’s interactions, IQ, and attentional development. We also excluded articles not in English or French, not focused on children below 3 years old or not focused on the effect of screens or touchscreens. We selected among the articles those that were related to the review’s topics: (1) the prevalence, correlates and screen viewing patterns, (2) screen viewing as a source of learning, (3) the effects of screen viewing on language development, executive functions, imitation, parent’s interactions, IQ, and attentional development, (4) the effects of viewing context, and (5) the causality in the effects of screen viewing on cognition. During this step, each of the authors carefully read the relevant articles for one or more sections he/she was in charge of and reviewed them in a narrative way. Additional related articles could be added. As a narrative review, we are highlighting only a subpart of the literature that is not necessarily representative of the whole field, but that we think can help to understand apparent contradictions in the literature.

We will start by showing the prevalence of screen exposure in infants (for both interactive and non-interactive screens), then we will review the effects of screen exposure on cognitive development, and of the different contexts of viewing on infants’ development, before discussing causal effects. We will end up with a discussion on the potential effects of screen exposure and the early development of cognitive abilities and communication.

## Prevalence, correlates and viewing patterns

The prevalence of exposure to screens in infants aged between 0 and 3 years has been the subject of many surveys in western countries, most of them conducted with North American populations and some with Europeans. More recent studies investigated the use of interactive screens in young infants specifically.

A recent large study conducted with a French population shows that 84% of 2-year-old toddlers watch television at least once a week, and 68% every day (Gassama et al., 2018). The average time of exposure to television for 2-to-24-month-old infants is 40 min per day and only half of the programs are educational programs, according to the parents (Zimmerman et al., 2007b). Moreover, in a cohort of children aged 6–18 months (Barr et al., 2010a), younger children were more exposed to adult programs than older. This suggests that infants are exposed both to infant- and adult- directed television. They typically attend 50% of the time only (Anderson and Pempek, 2005). These findings are particularly relevant to early cognitive development, as adult-directed content may be detrimental to play, language development and executive functioning, particularly for young infants, as we will see later.

As for interactive screens, a recent French survey shows that roughly 30% of 5-month-old infants use touchscreens and this percentage increases to 90% at 2 years (Cristia and Seidl, 2015). Frequency of exposure did not increase with age and between 5 and 24 months, 21% of infants used touchscreens daily, 32% weekly and 48% less than once a month. Another large, recent French survey showed similar results with 21% to 28% of 2-year-old children playing with a touchpad, a computer or a smartphone at least once a week and 10% to 12% of toddlers doing so daily (Gassama et al., 2018). These percentages are very close to those of the Common Sense Media study with an American sample (Rideout and Robb, 2020). These results suggest that just like television exposure, interactive screen use is present very early on in development and represents a significant time in some infants’ daily activities.

More studies are needed particularly for interactive screen use in order to understand whether environmental factors can influence these figures. For example, Kabali et al. (2015) reported even higher rates of use of interactive screens in a sample of low-income minority children, with 75% use among those between 12–36 months. The environmental factors associated with exposure to television have been more documented. For example, it varies according to the type of childcare. The majority of the time spent watching television (about 3 h daily) occurs at home in the presence of parents (Christakis and Garrison, 2009; Tandon et al., 2011). In non-parental childcare, the time spent in front of the television is shorter when in daycare (about 10 min daily) and greater when the care is at the child’s home (1.5 h daily), and is negatively correlated with the caregiver’s level of education (Christakis and Garrison, 2009).

Why are parents increasingly exposing their children to screens? The motivations that parents report for using television are varied (Garrison and Christakis, 2005; Linebarger and Walker, 2005; Zimmerman et al., 2007b): its used as a nanny (21%), the belief that programs are entertaining for infants, its use as a means of relaxation (23%), and as an educational tool (29%). As for parents’ attitudes toward the use of interactive screens include learning, creativity, entertainment, and soothing when distressed (Radesky et al., 2014, 2016; Nevski and Siibak, 2016; Levine et al., 2019; Dardanou et al., 2020).

In conclusion, even though these studies are mainly based on parents’ reports and do not prove causation, they show that interactive and non-interactive screens are becoming more pervasive in early childhood. The effects and consequences of that screen time exposure have received considerable attention in research over the past decade and enough work now exists to address the question of the effect of exposure for children younger than 3. In the next sections, we will review experimental research on how infants retain information from screens and then present correlational studies that investigate the effects of screen viewing on cognitive development.

## What kind of information can infants process through screens?

Before reviewing in detail the effects of watching screens on cognitive development, we would like to discuss how young children make use of information presented to them on screens and how they learn from videos.

An important perceptual difference between reality and screens is that reality is perceived in depth through stereoscopic vision, whereby the two separate images captured by each eye are combined by the brain. Stereoscopic vision develops around 5 months of age (Takai et al., 2005) although it remains very poor for years, and pictorial depth perception, the ability to perceive depth in 2D images, emerges around 7 months of age and continues to develop during the first 2 years (Yonas et al., 1978). Standard screens do not contain stereoscopic information and screens also differ from reality in other aspects: their luminance is lower, they cover a smaller field of view and some of them cannot be interacted with. These perceptual differences may interfere with infants’ ability to learn from videos or to generalize from the screen to the real world. By 6 months of age, infants can reproduce new actions directed at objects shown on a screen, actions that they would otherwise not produce spontaneously, after simply manipulating the objects (Meltzoff, 1988; Barr and Hayne, 1999; Hayne et al., 2003; Barr et al., 2007a,2010b; Barr and Wyss, 2008; Strouse and Troseth, 2008). At this age, a video model yields the same level of imitation as a live model (Barr et al., 2007a). However, by 12 months, it takes twice as many demonstrations (Barr et al., 2007b) and exposure time (Strouse and Troseth, 2008) for infants to imitate actions from a 2D model on screen than from a real 3D model. Thus, whilst young infants may be able to reproduce actions they saw on a screen, overall, they do not seem to view video as relevant to real life. This effect is called the “video deficit effect.” The perceptual impoverishment hypothesis suggests that the deficit is a result of the poorer stimulation on screens when compared to the real world (Barr and Hayne, 1999).

Many studies have explored how infants associate information from TV screens with real objects (Troseth and DeLoache, 1998; Troseth, 2003; Deocampo and Hudson, 2005; Troseth et al., 2006; Krcmar et al., 2007) or generalize information to the real world when it is learned from a touchscreen (Zack et al., 2009). In general, these studies show that 15–24 months old infants have difficulties generalizing an action learned on a TV screen to a real situation and vice versa, or to locate an object in the room when clues are given through a screen. Children also imitate the adult more when the on-screen model interacts in real time with the child than when the model is filmed in advance and cannot interact with him/her. Children can indeed locate an object in the room using clues provided by the adult interacting on the screen (Troseth et al., 2006): interaction with others remains a privileged source of learning and information.

By the age of 24 months, children start looking for different durations at *Teletubbies* when it is presented with backwards speech (each utterance is run backwards although occupying the same video frames) rather than with normal speech (Pempek et al., 2010). Therefore, it is not clear that infants can understand speech from video before the age of 2. There is anecdotal evidence that toddlers can learn words from watching television (Rice, 1983). More ecological studies (DeLoache et al., 2010) showed that learning new words through educational videos is negligible between 12 and 18 months of age. Infants were asked to point to objects while they were listening to the names of these objects, either from a video or by interacting with the parents. Infants did not learn any words in the video condition, unlike the adult-interaction condition, despite that these videos were considered very educational by the parents. Note that the context is important: the narration of the action favors its imitation (Seehagen and Herbert, 2010; Simcock et al., 2011). Thus, the percentage of children who imitate increases considerably when the objects presented on screen are named or commented by the parents or by the video, compared to presentations without parental comment or support (Barr and Wyss, 2008).

Finally, we would like to open a methodological discussion on the use of screens during experiments in laboratories studying infant’s behavior (Esseily et al., 2017). Given what infants perceive on a screen, how does it affect experimental conclusions? For example, when using the preferential looking paradigm, some conclusions might not generalize to real life stimuli, given the video deficit effect.

To summarize, learning from screens in infants appears to be negligible without parental or adult guidance, mainly because of the video deficit effect and difficulties to process speech on video. How does it affect the development of language?

## The effects of screens on the development of language

The relationship between the effects of watching screens and the development of children’s cognitive skills is complex as the time spent viewing screens *per se* is only one factor among others. We start by reviewing correlational studies showing the effects associated with screen time on language development. Later, we will review the factors modulating the effects associated with screen exposure on language, attention, executive functions, adult interactions and school readiness.

The link between screen viewing and language development is one of the most explored in the literature. It is clear that language learning takes place in an active way and that interactions play a primary role in it (Bruner, 2011). However, television viewing is generally non-interactive, except for programs specifically designed for interaction, therefore one can expect deficits in language development from over-exposure to television.

Indeed, 2 h a day spent watching television between 15- and 48-months of age multiplied by four the probability of a delay in language development. This delay was multiplied by six when children started watching television before 12-months (Chonchaiya and Pruksananonda, 2008). In this case-control study, the authors also evidenced that children at age 2 who had language delay usually started watching television earlier than a control group, and also spent more time watching television than other children (around 3 h per day vs. less than 2 h per day). Children who started watching television during their first year and who watched television more than 2 h/day were approximately six times more likely to have language delays than the ones who did not. Lin et al. (2015) also evidenced that children who were exposed to television 1 h daily before the age of 2 had an increased risk of delayed language development. Furthermore, the amount of time spent watching television alone before the age of 3 was associated with poorer syntax levels at ages 3 and 4 (Naigles and Mayeux, 2001). In addition, 6-month-old children exposed to television for an average of 2 h per day had poorer cognitive performances and lower language levels at 14 months of age than unexposed children (Tomopoulos et al., 2010). Zimmerman et al. (2007a) tested the association of media exposure with language development in children under the age of 2. Parents were asked to assess their child’s vocabulary through the short form of the MacArthur-Bates Communicative Development Inventory (CDI). Among infants (ages 8 to 16 months), each hour per day of viewing infant-directed DVDs/videos was associated with a decrease in CDI scores in a fully adjusted model. In older toddlers (ages 17 to 24 months), there were no significant associations between any type of media exposure and CDI scores.

Nonetheless, other authors (Ferguson and Donnellan, 2014) reanalyzed Zimmerman et al. (2007a)’s dataset and showed that opposite conclusions could be drawn depending on the chosen statistical analysis. For one of them, infants exposed to no screen actually had lower levels of language development compared to infants with some exposure. This highlights recent concerns over methodological degrees of freedom and the possibility of increased false positives in the psychological literature. It is also possible that other studies exist with the same conclusions but that could not be published because of non-significant results.

One possibility to explain the negative effects is that young children have reduced interactions with adults while watching television. This point seems important, as interactions are known to be the core format for language development in young children (Bruner, 2011). Another possibility is that the programs children were exposed to in these studies were produced for adults (Zimmerman and Christakis, 2005). Because children of this age pay little overt attention to such programs and likely have little comprehension of them, adult programing can be considered background television from the perspective of the child. Overall, this particular context in which children watch adults’ programs on television seems to reduce the quantity and quality of parental language addressed to their 12- and 24-month-old children (Christakis et al., 2009; Pempek et al., 2014). These aspects will be discussed in the next section.

As a summary, studies investigating the association between the amount of screen viewing and language development, without differentiating between child and adult programs viewed, found an overall negative association in children younger than 3. However, the amount of viewing does not seem to be the most important factor to consider. In recent years, evidence was provided that the focus should be on the quality (or context) of viewing, not the quantity.

## The importance of the context of viewing

A factor analysis of viewing patterns in bilingual toddlers (Hudon et al., 2013) extracted two factors having opposite effects on language development: quantity and quality. Quantity of viewing was not correlated with language outcomes, but poor quality was related to lower vocabulary. Poor quality was defined as television unintended for children, background television, solitary viewing, and earlier age of viewing.

Inspired by these results, we define the context of viewing as four aspects that modulate the effects of screens on cognitive development: (1) the type of content viewed and its structure, depending on the child’s age, (2) the caregiver’s behavior during viewing, (3) whether the program is watched or in the background while doing something else, and (4) the screen interactivity.

### The type of content viewed and the content structure

It is important to distinguish between the effects of exposure to contents created specifically for infants and young children and those intended for an adult audience (Anderson and Pempek, 2005). Below, we review how the type of content modulates the effects of screen viewing on school readiness, executive functions, attention skills, child-adult interactions and language development.

Regarding school readiness, Wright et al. (2001) collected time-use diaries of television viewing and found that 2-year-olds who were exposed more to child-directed educational programing, such as *Sesame Street*, reached higher scores on general measures of school readiness (knowledge of letters, numbers, colors, shape, spatial and size relations) at ages 3 and 4, than those who were primarily exposed to adult directed television programs. Conversely, heavy viewing of general-audience programs at age 2 predicted poorer performance on measures of mathematical skills and receptive vocabulary.

Regarding executive functions, screen exposure at 4 months was related to worse inhibitory control 10 months later, controlling for covariates through propensity scores, though there was no association between screen exposure and working memory or cognitive flexibility in this parental report study (McHarg et al., 2020a). Furthermore, screen exposure at 24 months was negatively associated with the development of executive functions from 24 to 36 months (McHarg et al., 2020b). Nevertheless, when looking at the content watched, a different picture emerged. Executive functions were reduced by exposure to programs aimed at adults when compared to programs aimed at children (Linebarger and Walker, 2005). Indeed, children who had higher levels of exposure to adult-directed television programs during infancy were rated by their parents as worse on executive functioning skills, like inhibitory self-control at age 4, in comparison to children who had lower levels of exposure (Barr et al., 2010c). On the contrary, early exposure to child-directed content was not associated with cognitive ability at age 4. Along similar lines, exposure to educational programs before age 3 was not linked to attention issues when reaching age 7 (Zimmerman and Christakis, 2007), while exposure to adult television content was negatively associated with executive functioning and cognitive skills at older ages (Christakis et al., 2004; Zimmerman and Christakis, 2005; Landhuis et al., 2007).

Concerning attentional skills, the number of hours of television watched daily at ages 1 and 3 predicted measures of hyperactivity at age 7, according to a large longitudinal survey (Christakis et al., 2004). However, children of different ages do not pay attention to the same types of content: looking time to child-directed programs is high, averaging approximately 70% for 12- to 18-month-olds (Barr et al., 2008) as these programs often have very dense perceptually salient features (Huston et al., 1981), which facilitates and scaffold comprehension of the content (Calvert et al., 1982). Thus, some educational programs designed for young children may well be beneficial while others indifferent or, in the case of adult programs, detrimental for cognitive outcomes. Indeed, educational television watched before age 3 was not associated with later attentional problems, while each hour of entertainment television was associated with doubled odds of attention problems, after adjusting for covariates (Zimmerman and Christakis, 2007).

Regarding language development, watching adult programs vs. child-directed programs between 15- and 48-months of age multiplied by 3 the probability of delaying language acquisition (Chonchaiya and Pruksananonda, 2008). More specifically, a population survey analyzed the characteristics of the content watched by two groups of 18-month-old children, one with delayed language development (Okuma and Tanimura, 2009). The delayed-language group watched more “detailed realistic animations” (like *Pinocchio* or *Spirited Away*) and “baby education” (e.g., videos teaching vocabulary) than the other. Their videos contained less close-ups of faces, more uninterrupted stories with constant movement or transformation of characters, had a higher frame rate, and adults readily kept on watching these videos even with the sound off. Another study using parental reports (Linebarger and Walker, 2005) found higher levels of language associated with watching programs containing a strong narrative and characters that address the child directly, providing pauses for the child to respond (e.g., *Dora the Explorer*). On the other hand, watching programs that show a loose narrative and contain complex stimuli (e.g., *Teletubbies*) is associated with poor language skills in children.

As a summary, the content of the videos is critical. Adult programs yield negative effects on the development of cognition before the age of 3, while child-directed programs are associated with either a positive effect or no effect. Furthermore, child-adult interactions are also affected differently, with less interactions during adult programs than child-directed programs (Mendelsohn et al., 2008). In the section about language development, it was also clear that interactivity with adults was a key factor to unlock the positive impacts of screen viewing in young children. Therefore, in the next section, we explore whether the parent’s behavior plays a role in modulating the effects of screen viewing on cognitive development.

### The caregiver’s behavior during viewing

As early as 6 months of age, having a parent who participates and comments on television program content has a positive effect on the child’s attention, as quasi-experimental studies show (Barr et al., 2008; Fidler et al., 2010). Indeed, the presence vs. absence of interactions during television viewing between 15- and 48-months of age modulates by 8 the probability of a delay in language development (Chonchaiya and Pruksananonda, 2008). Educational programs can also form a basis for play and creativity between parents and babies in the first 2 years of life, for example encouraging parents to name objects, in *Baby Einstein*, or to imagine new activities, in *Sesame Stree*t (Pempek et al., 2011). Whereas parents speak less to their infants during co-viewing of infant-directed television programs (compared with no television), they also tend to use richer vocabularies both during and immediately after viewing (Lavigne et al., 2015).

Although there have been no comparable studies on the impact of interactive screen media during infancy, there is some experimental evidence that toddlers (24 to 36-month old) can more readily learn from touchscreen devices than they can from television (Kirkorian et al., 2016). However, mobile devices have been shown to considerably reduce parental interactions with young children (Radesky et al., 2014). Thus, it appears that both television and interactive media may reduce at least the quantity of parent-child interactions, which are crucial for the development of cognitive skills, especially language and executive function. In addition, a telephone survey showed that only 32% of parents say that they watch television with their children (Zimmerman et al., 2007b).

In these studies, television is always the direct attentional target of the child and parents. But what happens when the television is turned on in the living room where children are present without specifically focusing on the screen, which creates background noise for all ongoing activities?

### The effect of television in the background

Background television can refer to two situations: (1) when the television is switched on in the background while the child is participating in other activities, (2) when a very young child is in front of or in the immediate vicinity of an adult program on a screen (Anderson and Evans, 2001). In the latter case, infants do not process information presented on screens for more than 3–5 s (for a summary, see Kirkorian et al., 2017), and have trouble processing speech (adult- and infant-directed speech) on screen until the age of 2 (Pempek et al., 2010; Anderson and Subrahmanyam, 2017; Hipp et al., 2017). The foreground screen becomes similar to a screen in the background and it is difficult to disentangle the effects of adult programs from the effects of the screen in the background. We have already reviewed the effects of adult programs in the section about content type and we will now summarize the results of the few studies that have more directly explored the effects of television in the background on the cognitive development of children under 3 years of age. We should note that exposure to background screens is more applicable to television than to mobile media, as the nature of mobile devices usually requires active engagement.

The consequences of early exposure to television in the background are twofold. On the one hand, the quantity and quality of parent-child interactions are affected, on the other hand, children are distracted from their ongoing activity.

Indeed, experimental findings show that parents talk less to 12- and 24-month-old children, and more passively, when the television is in the background than when it is turned off (Kirkorian et al., 2009). Questionnaire-based data also show that mothers use less vocabulary while playing with their 13-month-old child when the television is switched on vs. off (Masur et al., 2016). The decrease mediates the negative impact of screens on the lexicon size of these children at 17 months. This is of importance considering that the number of words heard before the age of 3 is a good predictor of future cognitive and linguistic performance (Hart and Risley, 1995; Risley and Hart, 2006; Zimmerman et al., 2009).

Another issue associated with background television is that it distracts the child from the action in progress, diverting their attention from play and learning. Experimental studies (Schmidt et al., 2008; Kirkorian et al., 2009; Setliff and Courage, 2011) have shown that television in the background interrupts the play sessions of children at 6, 12, 24, and 26 months of age. Even if children do not watch the screen much (5% of the time), the audiovisual changes that frequently occur on television cause the child to repeatedly orient toward the screen, draining the cognitive resources necessary to instantiate and execute action schemes. Advertisements in particular attract children’s attention because young children still have little control over their attentional focus (Ruff and Rothbart, 2001). Studies that looked at the quality of play reveal shorter play episodes, and shorter periods of focused attention in the presence of television in the background, resulting in less rich and less complex solitary play compared to when the television is off (Schmidt et al., 2008; Kirkorian et al., 2009; Masur et al., 2016).

We have seen the importance of sustained interactions between child and adult and how screens can decrease these interactions, even when in the background. Nowadays though, touchscreens have opened the doors to more interactivity. Could interactive screens replace to our advantage some of these interactions with the adult, or be used to enrich them, for the benefit of the child?

### The effect of interactive screens on learning

Here, we review the effects of interactive screens on cognitive development. This section does not include contingent communication through screens which is another area of research. A recent meta-analysis (Xie et al., 2018) showed that young children (0 to 5) could learn from touchscreens. When children were physically interacting with the screens, they learned better than other groups, like traditional classroom teaching, or could learn from video chats (Roseberry et al., 2014; Myers et al., 2017; Strouse et al., 2018). However, the effect of interactive screens with young infants is complex and depends on several factors, like age, content or comparison group. For example, older children learned better from touchscreens than younger ones; however younger ones learned better when the content was related to science as opposed to other material such as language comprehension, and when compared to non-interactive videos than when compared to manipulating physical objects.

Infants can learn from touchscreens, but can they transfer what they have learnt to real objects? Indeed, 15-month-old infants who have learnt from touchscreens only transferred their learning to touchscreens, and those who have learnt from real scenes only transferred their learning to real scenes, arguing for a video deficit effect extending to touchscreens, as experimental studies show (Zack et al., 2009; Barr, 2013). However, this deficit effect can be overcome through contingent communication with an experimenter on a screen. Indeed, 2- to 3-year-old children can learn new words and use clues given by an experimenter on a screen to find a hidden object only when the experimenter on the screen is interacting with the infant (Lauricella et al., 2010; Strouse and Ganea, 2017; Troseth et al., 2018). Other studies show a developmental trend between 24 and 30-months of age where younger infants do not learn from a touchscreen without interaction with a live partner, whereas 30-month-olds can learn without (Kirkorian et al., 2016). The authors argue that interactive videos may facilitate learning by directing attention to relevant information, thereby supporting limited attention skills that otherwise might rely on bottom-up, stimulus-driven features (Frank et al., 2009; Kirkorian et al., 2012).

Studies investigating the context of learning show that interaction with the parents enhances learning from touchscreen and transfer between-dimensions: infants were 19 times more likely to succeed and transfer learning between the touchscreen and real object if they were in a high-quality interactional dyad during a semi-naturalistic teaching task (Zack and Barr, 2016). The importance of the adult’s role in accompanying their child when interacting with a screen was also observed using a word-learning app (Walter-Laager et al., 2017). Infants who were accompanied by an adult had the largest growth in vocabulary, and those who used the word-learning app without adult accompaniment showed the second largest growth. Less successful were the children who played with the picture cards (with or without adult accompaniment). Social facilitation was also observed with peers: the presence of a 9-month-old peer increased vocabulary learning through a touchscreen (Lytle et al., 2018). Authors suggest that the presence of similarly aged peers may have increased their arousal and motivation to learn as they showed more vocalization than when alone with their caregiver. In addition, the authors found a positive correlation between learning and the number of new infant peers were paired with through trials arguing that novelty heightens arousal and may thus have enhanced learning.

At the moment though, it is not clear whether interactive screens disrupt social interactions like other types of screens, or on the contrary, if they support social interactions. Studies on electronic books and reading comprehension in young infants might bring some answers to this question. However, the existing studies show opposite results, some showing a positive effect of electronic books in engaging children in the story and in the interaction with the parent compared to classic paper books (Strouse and Ganea, 2017) and others showing negative effects, with less dialogic verbalizations from parents and less engagement from infants with electronic books (Strouse and Ganea, 2017; Munzer et al., 2019). Lastly, studies focusing on very young infants before 2-years of age are also scarce and would be necessary in order to understand the implications of early use of interactive screens.

## Is there a causality in the effects of watching screens on cognition?

Establishing causality relationships in science can be challenging, especially when experimental evidence cannot be collected for ethical reasons, as in the case of the potential harming effect of screen viewing on very young children. In that regard, we followed Suppes’ probabilistic theory of causality: “one event is the cause of another if the appearance of the first event is followed with a high probability by the appearance of the second, and there is no third event that we can use to factor out the probability relationship between the first and second events” (Suppes, 1970, p10). In other words, if one can establish a statistical association between two variables, show evidence of directionality and rule out the effects of likely confounding variables, one can build a solid case in favor of causality.

The majority of the studies cited in this article are cross-sectional studies establishing an association between screen viewing and cognitive development but no directionality. While it is possible that watching screens has a negative causal effect on attention, for example, it may very well be that toddlers with attention control problems are less likely to refrain from watching screens. It is also possible that the parents of toddlers with attention control issues use the screens more often as a nanny, in which case viewing is not the cause of attention control disorders but a consequence.

A couple of studies have investigated directionality between these variables for children below 3. A path analysis (Wright et al., 2001) revealed that more time spent watching children’s educational programs during age 2 and 3 predicts better reading, mathematics, receptive vocabulary, and school readiness scores 1 year later, while children scores did not predict educational screen time 1 year later. On the contrary, the time spent watching non-education children programs or programs not intended for children generally predicts lower scores 1 year later, but some of the effect can be explained by the fact that lower scores generally predict more watching of content not intended for children 1 year later. However, for an unknown reason, the authors used the scores between ages 3 and 4 to predict viewing patterns 1 year later rather than the scores between ages 2 and 3. A similar statistical method estimating the directional effects that one variable has on another at different timepoints (random-intercepts, cross-lagged panel model) was applied on a large cohort of children assessed at ages 2 and 3, using a parent-report scale to measure cognitive development (ASQ-3) (Madigan et al., 2019). Children’s screen time at 2 was linked to lower cognitive scores at 3, but the reverse was not true, indicating precedence of screen time on cognitive development in children younger than 3. The effect size was small, equivalent to a loss of 0.06 to 0.08 standard deviation for every daily hour spent in front of a screen (Guez and Ramus, 2019). In this study, screen time encompasses television but also active screen usages like video gaming that is known to have causal positive effects on cognition in school-age children (Franceschini et al., 2013; Gambacorta et al., 2018; Franceschini and Bertoni, 2019).

Interestingly, the two studies also spent significant efforts to factor out the effect of other likely variables. The first study ruled out the effect of important demographic factors through the Home Observational Measure of the Environment score and the language used at home (Wright et al., 2001). The second study also ruled out important demographic factors, including sleep time (Madigan et al., 2019). Indeed, even if screens have a causal effect on cognitive development, such an effect can still be indirectly mediated through another variable, for example, through a change in the child’s sleep pattern. Television and touchscreen use have been associated with a decrease in sleep quality (Cheung et al., 2017), and reduced night sleep (Ribner et al., 2019) in children younger than 3. An alternative mediation hypothesis is that watching screens does not have a direct detrimental effect but distracts children from other important daily engagement in play or learning with others (Kucirkova and Zuckerman, 2017). More research is needed to understand the precise causal structure through which screen watching can affect cognition in infants. At the moment, we can state that at least two studies established a solid case in favor of some causality of screen viewing on the toddler’s cognition. While the debate is still open, only experimental research can bridge the remaining gap to a definitive answer.

## Conclusion

In this review, we mainly focus on the potential impacts of early screen exposure on the development of cognitive abilities, but there might be other impacts on health and physical developmental associated with early screen exposure (e.g., sleep, physical activity, motor development) that we do not discuss.

From our review, it is clear that (1) interactive and non-interactive screens are becoming more pervasive in early childhood (Gassama et al., 2018); (2) between 12 and 30 months of age, there is a video deficit effect, for interactive and non-interactive screens (Barr, 2013), and until age 2, infants have trouble understanding speech on screens without adult guidance (Pempek et al., 2010). It helps explain why infants learn less from screens than from the real model, and generalize less the information on screens; (3) screen viewing is associated with lower cognitive development when viewing is unsupervised, when content is not appropriate for the age, or when in the background (Kirkorian et al., 2009); (4) therefore, it is not watching screens *per se* that determines the effects on development but rather the viewing context. Indeed, supervised viewing of appropriate-age content in the foreground can be beneficial, particularly when interactions occur; and (5) the effect of screens is likely causal (Madigan et al., 2019) but more work is needed in that respect. Screen viewing in the wrong context mainly impairs language development, school readiness, executive functions, attention capacities and parent-child interactions (Wright et al., 2001; Chonchaiya and Pruksananonda, 2008; Kirkorian et al., 2009; McHarg et al., 2020b).

There are at least two routes through which watching screens can have deleterious or beneficial effects on development. The first is linked to the inappropriateness of the program structure for the young child. Weak narrative, fast pace and editing, complex stimuli, or stimuli too different from reality, can make it difficult for the child to extract or generalize information. However, when screen content is appropriate for the child’s age, it can be beneficial, or have no detrimental effect, particularly when the content is designed to foster child’s interactions (Linebarger and Walker, 2005).

The second route is that viewing time may replace more appropriate learning activities, like social interactions. Interactions are also decreased during adult programs and when the screen is in the background (Kirkorian et al., 2009). Indeed, some studies evidenced that child-parent interactions were less communicative, and therefore less beneficial to the children, in presence of any types of screen exposure compared to other types of activities (e.g., books reading, playing with toys) and in the absence of screens (Nathanson and Rasmussen, 2011). It is therefore legitimate to question the effect of exposure to watching screens before the age of two, especially since exposure to screens is increasingly precocious (Wartella et al., 2010). However, when screens are used as a tool to support joint attention and adult-child interaction, they are beneficial (Fidler et al., 2010). Screens are impossible to remove from homes and are gradually making their way into school systems. It is imperative to inform caregivers of children younger than 3 about the risks associated with prolonged exposure to screen viewing in the wrong context and instead reinforce contexts that promote learning, such as viewing chosen age-adapted content and viewing with adult supervision.

One perspective for research is to develop more objective measures for screen viewing time in young children and to establish the reliability and validity of these measures. Current research mainly relies on parental reports. One possibility is to use media tracking apps from direct behavioral measures (eye-tracking) in the future. Further research is also necessary to distinguish between correlates for infants (under 12 months) and toddlers, as well as different kinds of media (television, mobile screens, touchscreens, video games) and media content. Existing research mostly focuses on one media at a time or no comparison is made between different media. Touchscreen media requires further attention, to assess for instance the effectiveness of specific touchscreen apps on children’s cognitive development. These could be developed to inform the efforts of parents, educators, and policymakers.

The associations of several environmental and contextual correlates with screen time still need to be clarified (e.g., maternal age, maternal education, household income). Further research could focus on clearly defining these factors and elucidating their role as well as the mechanisms by which they shape infant’s screen habits. Similarly, certain environmental and behavioral factors remain understudied, such as daily sleep duration, infant crying duration, or co-viewing habits. They may provide additional opportunities for intervention.

Viewing and using screens outside the home, in day care and pre-school settings adds to the total amount of time that children spend with screens and exposes them to additional, and perhaps different screen media contents that may lead to different developmental outcomes. Therefore, exploring screen media use in these settings and examining its impact on children’s development is also worthy of investigation.

## Author contributions

BG was in charge of harmonizing the text. AC was in charge of coordinating the work. All authors listed have made a substantial, direct, and intellectual contribution to the work, and approved it for publication.
